# Autophagy and Alzheimer’s Disease: Mechanisms and Impact Beyond the Brain

**DOI:** 10.3390/cells14120911

**Published:** 2025-06-16

**Authors:** Zaw Myo Hein, Thirupathirao Vishnumukkala, Barani Karikalan, Aisyah Alkatiri, Farida Hussan, Saravanan Jagadeesan, Mohd Amir Kamaruzzaman, Muhammad Danial Che Ramli, Che Mohd Nasril Che Mohd Nassir, Prarthana Kalerammana Gopalakrishna

**Affiliations:** 1 Department of Basic Medical Sciences, College of Medicine, Ajman University, Ajman P.O. Box 346, United Arab Emirates; z.hein@ajman.ac.ae; 2 Anatomy Discipline, Human Biology Department, School of Medicine, IMU University, Bukit Jalil, Kuala Lumpur 57000, Malaysia; thirupathirao@imu.edu.my (T.V.); 00000038358@student.imu.edu.my (A.A.); faridahussan@imu.edu.my (F.H.); 3 Department of Pathology, Faculty of Medicine, MAHSA University, Bandar Saujana Putra, Petaling Jaya 42610, Selangor, Malaysia; barani@mahsa.edu.my; 4 Department of Anatomy, School of Medicine, Lakeside Campus, Taylor’s University, Subang Jaya 47500, Selangor, Malaysia; saravanan.jagadeesan@taylors.edu.my; 5 Department of Anatomy, Faculty of Medicine, University Kebangsaan Malaysia, Jalan Yaacob Latif, Bandar Tun Razak, Cheras, Kuala Lumpur 56000, Malaysia; mohdamir@ukm.edu.my; 6 Faculty of Health and Life Sciences, Management and Science University, Seksyen 13, Shah Alam 40100, Selangor, Malaysia; muhddanial_cheramli@msu.edu.my; 7 Department of Anatomy and Physiology, Faculty of Medicine, University Sultan Zainal Abidin, Kuala Terengganu 20400, Terengganu, Malaysia; 8 Physiology Discipline, Human Biology Department, School of Medicine, IMU University, Bukit Jalil, Kuala Lumpur 57000, Malaysia

**Keywords:** Alzheimer’s disease, autophagy, amyloid-beta clearance, tau pathology, neurodegeneration

## Abstract

Alzheimer’s disease (AD) is a progressive neurodegenerative disorder marked by neuronal loss, cognitive decline, and pathological hallmarks such as amyloid-beta (Aβ) plaques and tau neurofibrillary tangles. Recent evidence highlights autophagy as a pivotal mechanism in cellular homeostasis, mediating the clearance of misfolded proteins and damaged organelles. However, impaired autophagy contributes significantly to AD pathogenesis by disrupting proteostasis, exacerbating neuroinflammation, and promoting synaptic dysfunction. This review aims to scrutinize the intricate relationship between autophagy dysfunction and AD progression, explaining key pathways including macroautophagy, chaperone-mediated autophagy (CMA), and selective autophagy processes such as mitophagy and aggrephagy. This further extends the discussion beyond the central nervous system, evaluating the role of hepatic autophagy in Aβ clearance and systemic metabolic regulation. An understanding of autophagy’s involvement in AD pathology via various mechanisms could give rise to a novel therapeutic strategy targeting autophagic modulation to mitigate disease progression in the future.

## 1. Introduction

Alzheimer’s disease (AD) is a progressive and irreversible neurodegenerative disorder primarily characterized by the destruction of neurons, leading to cognitive decline, memory loss, and changes in behavior and personality. It is the most common cause of dementia worldwide and represents a growing public health concern as global life expectancy increases. As of 2020, dementia, including Alzheimer-related forms, affects over 50 million people globally, with projections suggesting this number will rise to more than 152 million by 2050 [1]. In the United States alone, AD ranks as the sixth leading cause of death, affecting over 5 million individuals and incurring an estimated annual healthcare cost of 305 billion USD, which is expected to escalate to 1.1 trillion USD by 2050 [2].

In Asia, a region experiencing rapid demographic aging, the burden of AD is similarly mounting. For instance, in Malaysia, the prevalence of AD was estimated at 0.126% in 2020 and is projected to increase to 0.454% by 2050, amounting to over 260,000 affected individuals [3]. Comparable trends are observed in other Asian countries such as China [4,5] and Japan [6], highlighting the urgent need for regional as well as global strategies in AD management and research.

The hallmark neuropathological features of AD include extracellular neuritic plaques and intracellular neurofibrillary tangles. These are primarily composed of aggregated amyloid-beta (Aβ) peptides and hyperphosphorylated tau proteins, respectively [7]. The accumulation of Aβ is believed to trigger a cascade of pathological events such as tau aggregation, synaptic dysfunction, oxidative stress, mitochondrial impairment, and neuroinflammation, which, ultimately, lead to neuronal death and brain atrophy [8]. Despite advancements in understanding these mechanisms, the precise etiology and sequence of pathological changes in AD remain only partially understood. Current therapeutic options are mainly symptomatic and do not prevent disease progression, highlighting the need for further exploration of underlying cellular processes.

One such process is autophagy, a cellular degradation and recycling pathway essential for maintaining protein homeostasis and cellular health. Emerging evidence implies dysfunctional autophagy in the pathogenesis of various neurodegenerative disorders, including AD [9]. In neurons, impaired autophagy may contribute to the accumulation of toxic proteins such as Aβ and hyperphosphorylated tau, thereby exacerbating synaptic loss and neuronal degeneration [10]. Moreover, autophagy intersects key pathological features of AD, including mitochondrial dysfunction, oxidative stress, and inflammation [8].

This review aims to explore the intricate relationship between autophagy and AD pathogenesis. By examining recent advances in our understanding of autophagic dysfunction in AD, we seek to highlight potential molecular targets and therapeutic strategies. Furthermore, we consider whether other interrelated cellular systems may modulate these pathways, offering novel insights into the complex biology of AD and new directions for intervention.

### Methodology

This narrative review was conducted by systematically identifying and synthesizing relevant peer-reviewed literature on autophagy and Alzheimer’s disease from multiple electronic databases, including PubMed, Scopus, and Web of Science. Searches were performed using keywords such as “autophagy,” “Alzheimer’s disease,” “amyloid-beta,” “tau pathology,” “selective autophagy,” and “neurodegeneration”. Studies published in English for the last 10 years were included (with the exception of certain relevant literature from longer than 10 years), with emphasis on recent high-impact publications and original research, mechanistic studies, and comprehensive reviews. Articles were selected based on their relevance to the scope of this review, methodological rigor, and novelty in elucidating autophagy-related mechanisms in AD pathogenesis and therapeutic targeting. Additional sources were identified through manual screening of reference lists from key articles.

## 2. Overview of Autophagy

Autophagy is a crucial cellular process that plays a vital role in maintaining cellular homeostasis. It is a tightly controlled mechanism that eliminates damaged organelles and misfolded proteins, aiding in cellular survival and adaptation to stressful conditions. It involves autophagosomes, which are a formation of double-membraned vesicles engulfing cytoplasmic material for degradation, promoting energy balance and renewal [11]. Christian de Duve first used the term “autophagy” in the 1960s, and it has since grown to be a well-researched phenomenon with important applications in a range of physiological and pathological conditions [12]. By ensuring the elimination of harmful proteins, organelles, and intracellular pathogens, autophagy acts as a quality control system to preserve cellular integrity and stop the buildup of toxic substances [13]. However, the dysregulation of autophagy has been implicated in a variety of pathological conditions, including cancer, metabolic syndromes, and neurodegenerative diseases such as AD. Thus, it is a crucial protective mechanism in cells that particularly targets and destroys unwanted substances and also microorganisms [14]. The dysregulation of autophagy can result in several illnesses, including cancer, metabolic syndromes, and AD [15]. This study reviewed the intricate relationship between autophagy malfunction and neuronal death in the etiology of AD.

Moreover, autophagy is classified into three primary types based on the contents of the cell that are integrated into the lysosome for degradation, for example, chaperone-mediated autophagy (CMA), macroautophagy, and microautophagy [16]. In CMA, the breakdown of cytosolic proteins involves transporting them directly into lysosomes and the lysosomal lumen [17]. In microautophagy, cytoplasmic material is internalized into the lysosome through direct invagination of the lysosomal membrane [18]. In macroautophagy, degradable cytoplasmic contents are enclosed within subcellular double-membrane structures known as “autophagosomes” that convey cellular “waste” to lysosomes for breakdown [19].

### 2.1. Macroautophagy

**Figure 1:**
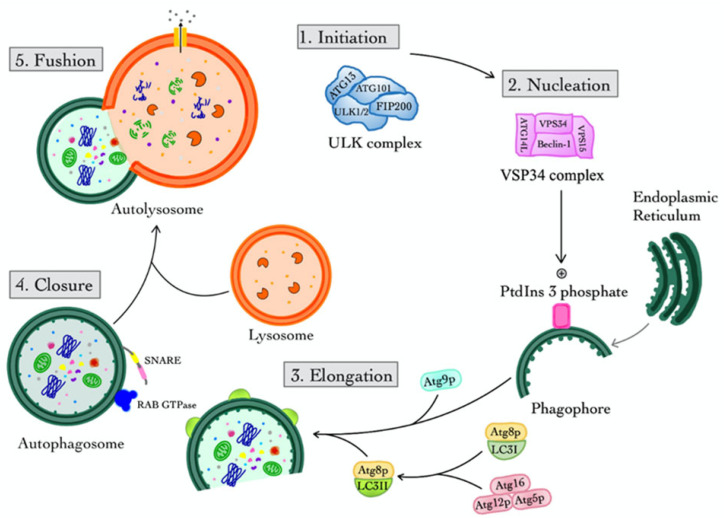
Schematic illustration of the five major stages of macroautophagy: (1) initiation begins with activation of the Unc-51-like autophagy-activating kinase (ULK) complex (ULK1/2, ATG13, ATG101, and FAK family kinase-interacting protein of 200 kDa or FIP200), triggering downstream events; (2) nucleation involves the vacuolar protein sorting 34 (VPS34) complex (including VPS34, VPS15, Beclin-1, and Atg14L) generating phosphatidylinositol 3-phosphate (PtdIns3P) at the phagophore formation site near the endoplasmic reticulum; (3) elongation is mediated by the conjugation systems involving Atg9p, Atg8p/LC3I/LC3II, Atg12–Atg5–Atg16L complex, expanding the phagophore membrane; (4) closure leads to the formation of a double-membraned autophagosome, facilitated by soluble N-ethylmaleimide-sensitive-factor attachment protein receptor (SNARE) proteins and RAB GTPases; and (5) fusion merges the autophagosome with a lysosome to form an autolysosome, enabling degradation of the enclosed cytoplasmic material. This pathway is essential for cellular homeostasis and the clearance of damaged organelles and proteins.

Macroautophagy is the primary and most thoroughly researched form of autophagy, often abbreviated as “autophagy.” The basal level of autophagy is low under physiological conditions; however, it can be rapidly induced by various stimuli, including energy deprivation, nutrient starvation, misfolded proteins, damaged organelles, infection, inflammation, and other stressors [20]. The primary event in autophagy is the formation of the autophagosome, which is facilitated by various proteins known as autophagy-related genes (ATGs). The autophagy process, referred to as autophagic flux, consists of four stages categorized by the status of autophagosomes: initiation, nucleation, elongation, closure, and fusion. Each step is precisely controlled by various molecules [21] (Figure 1).

#### 2.1.1. Initiation

The ULK complex, which includes ULK1, ULK2, ATG13, FIP200, and ATG101 in mammals, is primarily responsible for the initiation of autophagy. ULK1 is like ATG1 in yeast, which is a serine/threonine kinase that phosphorylates downstream proteins to signal autophagosome formation [22,23]. Moreover, the mechanistic target of rapamycin complex 1 (mTORC1) acts as a sensor for nutrients and energy, inhibiting the ULK complex in nutrient-rich situations. Whereby, in times of starvation or stress, the AMP-activated protein kinase (AMPK) phosphorylates ULK1 while suppressing mTORC1, enabling the start of autophagy. ATG101 stabilizes the ULK1/2-ATG13-FIP200 complex and boosts its kinase activity [24] (Figure 1).

#### 2.1.2. Nucleation

Nucleation is the formation of the phagophore assembly site (PAS) or isolation membrane, which signals the start of autophagic membrane creation (Figure 1). VPS34, Beclin 1 (BECN1), ATG14L, and p150/VPS15 are all necessary components of the Class III Phosphoinositide 3-Kinase (PI3K) complex [20]. In this case, VPS34 produces phosphatidylinositol 3-phosphate (PtdIns3P) at the PAS, which attracts PtdIns3P-binding proteins such as the WD repeat domain, phosphoinositide interacting 1 and 2 (WIPI1/2), and double FYVE-containing protein 1 (DFCP1), hence facilitating membrane nucleation. On the other hand, Beclin 1 is controlled by Bcl-2 family proteins, and Bcl-2 binding inhibits autophagy under normal conditions. Moreover, under stress, Bcl-2 dissociates to allow complex activation [20].

#### 2.1.3. Elongation

Elongation converts the phagophore into a double membrane autophagosome. Two significant ubiquitin-like conjugation pathways exist: firstly, the ATG12-ATG5-ATG16L1 complex that acts as an E3 ligase, promoting the lipidation of microtubule-associated protein 1A/1B light chain 3 (LC3). Secondly, the LC3/Atg8 conjugation pathway involves ATG4 cleaving LC3 to produce LC3-I, which is then conjugated to phosphatidyl-ethanolamine (PE) to form LC3-II and embedded in the autophagosomal membrane. Moreover, LC3-II is a marker for autophagosomes that help with membrane tethering, expansion, and cargo recognition. Moreover, ATG9/ATG9A vesicles provide additional membrane sources and are controlled by scramblase activity, which aids in lipid redistribution and membrane expansion (see Figure 1). WIPI interacts with ATG16L1, facilitating the recruitment of the LC3 lipidation machinery to the phagophore [25].

#### 2.1.4. Closure

The closure is the process by which the open autophagosome membrane seals, forming a complete vesicle. The endosomal sorting complex required for transport (ESCRT) process has been linked to the closure of phagophores. Moreover, the ATG2-WIPI complex is involved in both lipid transport and membrane closure. Additionally, syntaxin 17 (STX17) is found on mature autophagosomes and is required for the subsequent fusion process [26,27].

#### 2.1.5. Fusion

Finally, the acidic lysosomal environment activates hydrolases, which degrade the sequestered cytoplasmic contents, including damaged organelles and misfolded proteins. Lysosomal permeases release nutrients into the cytosol for recycling, including amino acids and fatty acids [28].

### 2.2. Selective Features and Regulation of Macroautophagy

Despite being characterized as a non-selective bulk degradation system, macroautophagy has highly regulated and selective mechanisms that allow for the targeted removal of certain cellular components via receptor-mediated processes. ATG proteins simplify cargo recognition, lipid conjugation, and membrane elongation. To ensure cargo capture and autophagy completion, autophagosome production is closely regulated [29,30,31].

The autophagy pathway breaks down certain cellular elements, or “cargoes,” called selective autophagy [32]. Selective autophagy involves designation, targeting, sequestration, and degradation. Designating distinct payloads with a molecular tag helps the autophagy machinery recognize them. A receptor directs payloads onto growing autophagosomes, which elongate and trap them. This is targeting and sequestration. Finally, the autophagosome merges with a vacuole/lysosome to break down cargo [32].

Moreover, autophagy cargo is assigned by multiple processes. In mammalian cells, ubiquitination sends a “eat me” signal to the central autophagic machinery [33]. These regulatory components can also remove cargo identification signals, like ubiquitination, to avoid autophagy [34]. Many mammals contain autophagy receptors, including p62 (also known as Sequestosome 1 or SQSTM1), NBR1, optineurin (OPTN), nuclear dot protein 52 kDa (NDP52), Tax-binding protein 1 (TAX1BP), and NIP3-like protein X (NIX) (also known as BCL2/adenovirus E1B 19 kDa protein-interacting protein 3-like or BNIP3L) [34]. Selectivity results from high-affinity connections between these receptors and cargo.

Additionally, the receptors use ubiquitin-binding domains and motifs to recognize and bind cargo, connecting with Atg8p/LC3 on autophagosomes through evolutionarily conserved patterns: the LIR in humans and the AIM in yeast to promote cargo sequestration [35]. Fusion with the lytic compartment degrades autophagy receptors. Recently, nuclear Dot Protein 52 kDa (NDP52) and OPTN have been found to directly engage with autophagy initiation machinery, attracting the ULK1-initiation complex to damaged mitochondria and cytosol-invading bacteria to degrade them [33,36,37].

## 3. Selective Autophagy

**Figure 2:**
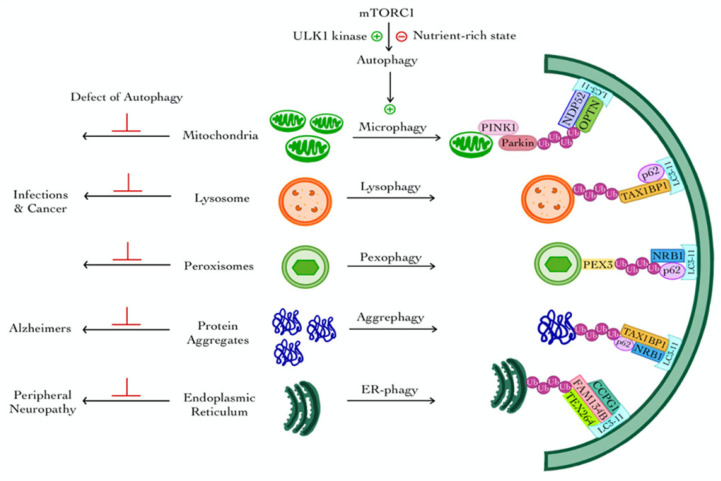
Schematic illustration of selective autophagy pathways and their implications in human diseases. Selective autophagy is activated under nutrient-deprived conditions through the inhibition of mechanistic target of rapamycin complex 1 (mTORC1) and activation of ULK1 kinase, leading to targeted degradation of specific organelles or protein aggregates. Various forms include the following: Mitophagy (mitochondrial clearance), regulated by PTEN-induced kinase 1/parkin (PINK1/Parkin), associated with neurodegeneration in autophagy-deficient states. Lysophagy (lysosomal turnover), critical in infections and cancer, is mediated by ubiquitination and autophagy receptors such as Tax1 (Human T-cell Leukemia Virus Type I) binding protein 1 (or TAX1BP1) and p62. Pexophagy (peroxisome degradation), involving peroxisomal biogenesis factor 3 (PEX3) and NRB1, affects metabolic disorders. Aggrephagy (clearance of protein aggregates), relevant to Alzheimer’s disease pathology, employing TAX1BP1, NRB1, and p62. ER-phagy (endoplasmic reticulum turnover), regulated by receptors like family with sequence similarity 134 member B (FAM134B) and cell cycle progression gene 1 (CCPG1), is implicated in peripheral neuropathy. Each process recruits LC3-interacting autophagy adaptors to facilitate engulfment by the autophagosome. Dysfunction in these selective autophagy pathways contributes to various human diseases.

Selective autophagy targets specific cargos for degradation and involves several pathways that are classified according to their target organelles: mitophagy (mitochondria), lysophagy (lysosomes), pexophagy (peroxisomes), aggrephagy (protein and RNA aggregates), and ER-phagy (endoplasmic reticulum) [26] (Figure 2).

### 3.1. Mitophagy

The selective removal of damaged mitochondria through the process of mitophagy is necessary to maintain the integrity of the cell. The most well-studied example of selective autophagy involves the PTEN-induced kinase 1 (PINK1)-Parkin mitophagy pathway, where functional mitochondria import PINK1 into the inner membrane, while dysfunctional ones accumulate PINK1 on the outer membrane [38,39]. Phospho-ubiquitin recruits mitophagy factors like OPTN and NDP52, while Parkin amplifies the signal by ubiquitinating mitochondrial outer membrane proteins. Designated mitochondria are targeted and sequestered within autophagosomes, allowing selective targeting of dysfunctional ones [40,41,42].

### 3.2. Lysophagy

Lysosomal membrane permeabilization, a common stress condition, is linked to degenerative diseases, infections, and cancer. Cells repair lysosomes using ESCRT machinery. When the repair is a failure, ubiquitin-tagged lysosomes begin the process of selective macroautophagy, or lysophagy, which leads to their removal [43]. This suggests that ESCRT-mediated repair and lysophagy are independent processes, clearing only unrepaired lysosomes. Selective autophagy involves autophagy receptors linking ubiquitinated cargo to the phagophore.

In a study involving L-leucyl-L-leucine methyl ester (LLOMe)-induced lysosomal damage, ubiquitination was shown to facilitate efficient lysophagy by recruiting autophagy receptors such as SQSTM1/p62 and TAX1BP1 [44]. Furthermore, optineurin is recruited to ubiquitin-tagged damaged lysosomes in the case of lysosomal damage by α-synuclein fibrils in neurodegenerative disorders [44]. Other studies also indicate that tank-binding kinase 1 (TBK1) collaborates with NDP52, an autophagy receptor, to prevent Salmonella-induced endomembrane damage, which recruits ULK1 and coordinates anti-bacterial autophagy [45]. This suggests that cells employ distinct autophagy receptors and regulation mechanisms for various types of endo-lysosomal membranes and damaging agents [45]. Moreover, according to recent literature, there are several new ubiquitination factors that attract autophagy machinery along with further layers of regulation, such as the processing of ubiquitinated proteins by the ATP-driven chaperone valosin-containing protein (AAA-ATPase VCP/p9) [43].

### 3.3. Pexophagy

Pexophagy is the selective autophagic degradation of damaged or excess peroxisomes, playing a crucial role in maintaining peroxisomal homeostasis. The mechanisms of pexophagy differ between yeast and mammalian cells. In yeast, the loss of peroxisomal AAA-type ATPases, specifically the Pex1p–Pex6p–Pex15p complex, renders peroxisomes import-incompetent, thereby promoting their degradation via pexophagy [46,47]. When key components of the peroxisomal protein import machinery are absent, ubiquitinated Pex5p accumulates on the peroxisomal membrane, signaling the organelle for selective degradation through specific autophagy receptors [46,47].

On the other hand, in mammalian cells, pexophagy is triggered by signals such as oxidative stress or excess peroxisome numbers [48]. The peroxisomal membrane protein PEX3 can promote pexophagy by recruiting autophagy receptors such as NBR1 and p62, which recognize ubiquitinated cargo and facilitate binding to LC3-II on the autophagosomal membrane. This interaction enables the sequestration and subsequent degradation of peroxisomes within the autolysosome [48].

### 3.4. Aggrephagy

Aggrephagy, a specialized form of selective autophagy, is responsible for the degradation of misfolded or aggregation-prone proteins. These protein aggregates are recognized and targeted by autophagy receptors such as TAX1BP1, NBR1, Tollip (Cue5 in yeast), OPTN, and p62. These receptors typically bind to ubiquitinated aggregates and facilitate their recruitment to the autophagic machinery for degradation [49]. mTORC1 plays a central role in regulating aggrephagy through its control over transcription factor EB (TFEB), a master regulator of lysosomal biogenesis and autophagy. Under nutrient-rich conditions, mTORC1 phosphorylates TFEB, retaining it on the lysosomal membrane and preventing its nuclear translocation [50]. Upon mTORC1 inhibition, either through nutrient deprivation or pharmacological intervention, TFEB becomes dephosphorylated and translocates to the nucleus. This activates the transcription of a network of genes involved in autophagy and lysosome formation, thereby promoting aggregate clearance through enhanced autophagic flux and lysosomal biogenesis [51].

### 3.5. ER-Phagy (Reticulophagy)

Autophagy plays a vital role in maintaining cellular homeostasis, and one of its specialized forms, ER-phagy (also known as reticulophagy), is essential for the selective degradation and remodeling of the endoplasmic reticulum (ER). ER-phagy is mediated by specific ER-resident receptors that recognize and target portions of the ER for encapsulation into autophagosomes. One key ER-phagy receptor is FAM134B, which localizes to the ER membrane and interacts with autophagy-related proteins such as LC3 and GABARAP. It also associates with reticulon 3, enabling the recognition and sequestration of specific ER regions destined for degradation. Additional ER-phagy receptors include CCPG1, Sec62, Atlastin-3, and testis-expressed 264 (TEX264), each contributing to the regulation of ER turnover in response to stress or cellular demands [52,53]. Mutations or dysregulation of these ER-phagy receptors have been implicated in various diseases, including hereditary sensory and autonomic neuropathies, underscoring the importance of ER-phagy in neuronal health and protein quality control [54].

The degradation of organelles like peroxisomes, in conjunction with selective autophagy, further exemplifies autophagy’s function in maintaining cellular homeostasis under various conditions. In addition to their role in lipid metabolism, peroxisomes are essential regulators of oxidative metabolism and cellular responses to hypoxia. Key transcription factors such as hypoxia-inducible factor-1α (HIF-1α) and HIF-2α mediate these adaptive responses [55,56,57,58]. Under hypoxic conditions, the inhibition of HIF-2α by the von Hippel–Lindau protein (VHL) is alleviated, leading to increased ubiquitination of PEX5, a peroxisomal import receptor, which, in turn, can initiate selective peroxisomal degradation [59]. Recent studies have also identified Tankyrase 1 (TNKS1) and Tankyrase 2 (TNKS2), members of the poly (ADP-ribose) polymerase (PARP) family, as novel regulators of non-canonical pexophagy, functioning through interaction with PEX14 to modulate peroxisome turnover. These mechanisms are also implicated in mitochondrial clearance, proinsulin granule degradation, and cellular iron homeostasis [60,61]. Consequently, like how ER-phagy focuses on the endoplasmic reticulum for quality assurance, pexophagy facilitates the degradation of peroxisomes, thus connecting autophagy to more extensive cellular reactions to stress and energy demands.

The multifunctional roles of autophagy-related (ATG) proteins extend beyond organelle-specific degradation, encompassing non-autophagic functions that enhance their influence on cellular homeostasis. For example, LC3-associated phagocytosis (LAP) plays a vital role in immune regulation and inflammatory responses in phagocytic cells. LAP is activated via Toll-like receptors (TLRs) and immunoglobulin receptors, facilitating LC3-associated endocytosis (LANDO), a pathway involved in the clearance of Aβ and the amelioration of AD pathology [62,63]. Additionally, components of the canonical autophagy machinery, including the ATG conjugation system, ULK1 complex, and PI3K–Beclin 1–VPS34 complex, are involved in various non-canonical autophagy pathways in a cell-type-specific manner [64,65]. The ubiquitin-proteasome system (UPS) further complements autophagic degradation by regulating protein turnover, with ubiquitin serving as a key signal for both proteasomal and autophagic clearance. Notably, p62 acts as a bridge between the UPS and autophagy. Proteasome inhibition has been shown to enhance nuclear translocation of autophagy regulators and activate compensatory autophagic pathways [66,67,68]. The varying actions of these ATG proteins and their interaction with other degradation systems underline the complex significance of autophagy in cellular health, linking organelle-specific processes such as ER-phagy and pexophagy to broader regulatory mechanisms.

While selective macroautophagy encapsulates certain cellular components in double-membrane vesicles, autophagy also exists in mechanistically separate forms that do not need autophagosome production. These encompass chaperone-mediated autophagy (CMA) and microautophagy, both of which directly carry substrates to the lysosome, highlighting the adaptability of autophagy in preserving cellular homeostasis in various environments.

## 4. Chaperone-Mediated Autophagy (CMA)

CMA is a selective form of autophagy in which specific substrate proteins are recognized and translocated directly into the lysosome for degradation. This process is initiated when substrate proteins containing a pentapeptide sequence [five amino acids: K (Lysine), F (Phenylalanine), E (Glutamic acid), R (Arginine), and Q (Glutamine)] or KFERQ-like motif are recognized by the cytosolic chaperone heat shock cognate 71-kDa protein (HSC70). The resulting HSC70–substrate complex binds to the cytosolic tail of lysosome-associated membrane protein type 2A (LAMP-2A) on the lysosomal membrane, triggering the multimerization of LAMP-2A into a functional translocation complex [69].

Once the substrate is engaged, it is unfolded and translocated into the lysosomal lumen, a process assisted by luminal HSC70. Following translocation, the LAMP-2A complex is disassembled to allow recycling of its components. Importantly, the rate-limiting step of CMA is the expression level and dynamic assembly/disassembly of LAMP-2A at the lysosomal membrane. Regulation of the LAMP-2A complex is mediated by a set of interacting factors, including lysosomal AKT, monomeric glial fibrillary acidic protein (GFAP), and elongation factor-1α (EF1α), which modulate complex stability in a GTP-dependent manner across all studied cell types [70].

**Figure 3:**
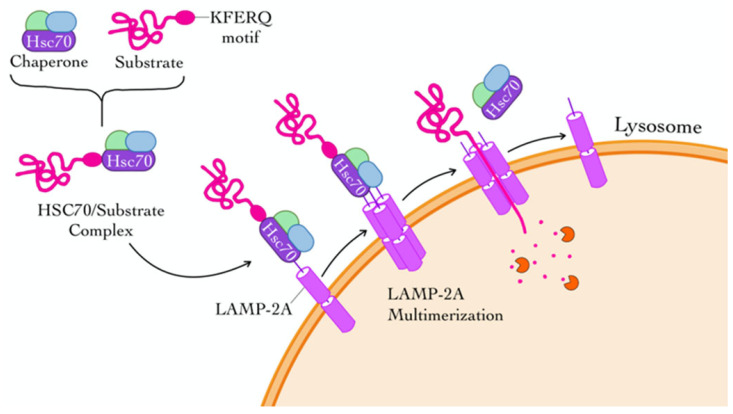
Schematic illustration of the steps involved in chaperone-mediated autophagy (CMA). Substrate proteins containing a KFERQ-like motif are recognized by the cytosolic chaperone heat shock cognate 71-kDa protein (HSC70), forming a substrate–HSC70 complex. This complex is then targeted to the lysosomal membrane, where it binds to the lysosome-associated membrane protein type 2A (LAMP-2A). Upon binding, LAMP-2A undergoes multimerization to form a translocation complex, facilitating the unfolding and transport of the substrate protein into the lysosomal lumen for degradation.

Phosphorylation of AKT by mTORC2 suppresses CMA activity, whereas dephosphorylation by PHLPP1 enhances CMA, leading to its maximal activation. CMA selectively targets a variety of proteins implicated in key cellular processes, including GFAP, PI3K, nuclear factor of activated T-cells (NFATs), nuclear factor erythroid 2-related factor 2 (NRF2), and tumor protein D52 (TPD52). Conversely, retinoic acid receptor alpha (RARα) and growth hormone signaling act as negative regulators of CMA [71,72] (Figure 3).

## 5. Microautophagy and Endosomal Microautophagy

Microautophagy is a form of autophagy where cytoplasmic components are directly engulfed by lysosomes or endosomes through membrane invagination or protrusion, facilitating their subsequent degradation [73]. A specialized subtype of this process in mammals, known as endosomal microautophagy, occurs within late endosomes/multivesicular bodies (LE/MVBs). The endosomal microautophagy targets soluble cytosolic proteins and can be further classified based on cargo selectivity and the molecular machinery involved [74].

Similar to CMA, one endosomal microautophagy pathway also recognizes proteins containing a KFERQ-like motif, which are bound by HSC70. These HSC70–substrate complexes are internalized into LE/MVBs via an endosomal sorting complexes required for transport (ESCRT)-dependent mechanism [75]. After internalization, lysosomal fusion with LE/MVBs enables the degradation of cargo proteins.

A distinct, HSC70-independent endosomal microautophagy pathway has also been identified in mammalian cells. This pathway still relies on ESCRT components but functions to degrade macroautophagy receptors, potentially playing a regulatory role in shifting the balance between selective and bulk macroautophagy [76]. These findings highlight the versatility and functional importance of endosomal microautophagy in maintaining proteostasis and fine-tuning autophagic responses under varying cellular conditions.

## 6. Autophagy in AD and Organ-Specific Impacts

### 6.1. Autophagic Impairment in Protein Aggregate Clearance

Mounting evidence implicates autophagy dysregulation as a critical contributor to the pathogenesis of AD. As early as 1967, Suzuki observed numerous aberrant subcellular vesicles and tau protein aggregates accumulating in dystrophic neurites in the brains of AD patients [77,78]. At the time, the identity of these vesicles was unknown. In 2005, Nixon and colleagues revealed that these vesicles were immature autophagic vacuoles (AVs), indicating a disruption in the autophagic pathway in AD [79,80].

Subsequent studies using AD mouse models and age-matched controls demonstrated that AVs begin to accumulate in neuronal dendrites and somata even before the appearance of extracellular Aβ plaques [81]. Notably, the aberrant accumulation of immature AVs in axons, particularly within hippocampal neurons, was observed well before detectable synaptic and neuronal loss, suggesting that impaired autophagic flux is an early event in AD pathogenesis [82]. Similar findings have been replicated in various animal models of AD, reinforcing the link between AVs accumulation and disease progression [83].

Moreover, autophagy plays a pivotal role in the clearance of pathological protein aggregates, including hyperphosphorylated tau. Studies have demonstrated that autophagic dysfunction, particularly autophagic gridlock, where AVs fail to mature and fuse with lysosomes, can exacerbate tau accumulation and contribute to the development of AD-like tauopathy [84,85]. Indeed, the abundance of autophagic vacuoles in AD brains, which is rarely observed in healthy controls, supports the hypothesis that impaired autophagy–lysosome function underlies the buildup of pathogenic proteins such as tau and Aβ [86].

Recent research has also highlighted the importance of mitophagy, a selective form of autophagy responsible for mitochondrial quality control, in AD. Impaired mitophagy contributes to the accumulation of dysfunctional mitochondria, leading to increased oxidative stress, energy deficits, and heightened neuronal vulnerability factors that further exacerbate AD pathology [86].

### 6.2. Lysosomal Dysfunction and Genetic Regulators

Amyloid precursor protein (APP), presenilin-1 (PS1), and presenilin-2 (PS2) are the key genes implicated in familial AD [87]. While mutations in PS1 and PS2 are known to increase Aβ production via their role in the γ-secretase complex, studies have shown that wild-type PS1 but not its mutated forms are also essential for lysosomal acidification, a function independent of its γ-secretase activity. This process is critical for maintaining the autophagy–lysosome degradation system [88].

In the case of sporadic AD, apolipoprotein E4 (ApoE4) represents the most significant genetic risk factor. ApoE4 has been found to disrupt autophagic function, contributing to disease pathology [89]. In transgenic mouse models expressing ApoE4, elevated levels of Aβ42 have been observed within lysosomes, leading to hippocampal neuronal death [90]. Additionally, in Neuro-2a cells, apoE4 exacerbates lysosomal membrane leakage and increases apoptosis triggered by Aβ peptides [91].

As discussed, a hallmark of AD is the progressive accumulation of AVs and lysosomal dysfunction in neurons. However, it remains debated whether this autophagic failure is a cause or consequence of AD pathogenesis [92,93]. Emerging evidence suggests that sex differences may also influence autophagy–lysosome system dysfunction in AD, although the mechanisms remain unclear [94]. Intriguingly, autophagy appears to play a neuroprotective role during the early stages of AD; however, in later stages, excessive or dysregulated autophagy may contribute to neuronal degeneration [95].

The earliest morphological evidence of autophagic impairment in AD is the abnormal accumulation of autophagosomes in neurons, particularly in hippocampal regions [81]. Despite decades of research, the exact mechanisms underlying autophagy failure in AD remain unresolved [96]. This failure may arise from one or more of the following: increased autophagy induction, impaired autophagic flux at the degradation stage, defective lysosomal function, or impaired fusion of autophagosomes with lysosomes [97]. Furthermore, lysosomal biogenesis is transcriptionally regulated by TFEB, a master regulator of autophagy and lysosomal gene expression. In AD, TFEB nuclear translocation is impaired, which hinders lysosomal renewal and functional degradation capacity [95]. Notably, enhancing TFEB activity in preclinical models has been shown to restore autophagic flux and reduce Aβ accumulation, offering a promising therapeutic avenue.

### 6.3. Autophagic Flux

Although both human AD brains and animal models exhibit significant accumulation of autophagosomes, this does not necessarily indicate enhanced autophagy activation [96]. In fact, the presence of accumulated autophagosomes may reflect impaired autophagic flux, where the degradation phase, particularly lysosomal fusion or clearance, is dysfunctional.

A key protein in autophagy initiation, Beclin-1, is found at reduced levels in neurons of AD patients when compared to healthy controls [84]. This reduction is believed to result from excessive caspase-3 activity, which cleaves Beclin-1, thereby attenuating autophagy initiation [98]. In transgenic APP mouse models with Beclin-1 deletion, intracellular Aβ accumulation increases significantly, and basal autophagic activity is disrupted, supporting the importance of Beclin-1 in AD pathology [99].

Additionally, the autophagic cargo receptor p62 (also known as SQSTM1), which binds to LC3 to facilitate cargo delivery to autophagosomes, has been evaluated in AD models. In a triple transgenic mouse model of AD, p62 expression was reported to be significantly reduced, potentially reflecting impaired autophagic cargo recognition or turnover [100]. However, other studies suggest an upregulation of autophagy in AD. For instance, a genome-wide transcriptomic analysis revealed that many positive regulators of autophagy are transcriptionally upregulated in AD, potentially as a compensatory response [101]. This may be driven by the reactive oxygen species (ROS)-mediated activation of class III PI3K and other autophagy-initiating kinases [102].

Interestingly, an increased autophagic flux has been reported in early-stage AD, particularly in CA1 hippocampal neurons [103]. In these stages, enhanced expression of ATGs, increased autophagosome formation, and elevated lysosomal biogenesis have been observed [104]. These findings suggest a biphasic pattern of autophagy regulation in AD, with initial upregulation followed by late-stage impairment. This concept is further supported by advanced imaging studies using tandem fluorescent-tagged LC3 reporters, which allow real-time visualization of autophagic flux in vivo [104]. These experiments demonstrate that early-stage AD is characterized by increased autophagosome production, whereas late-stage disease features autophagosome accumulation due to failed autophagosome-lysosome fusion and subsequent degradation deficits [104].

Given these conflicting findings, it is critical to develop stage-specific assessments of autophagy dynamics in AD. Autophagy may be subject to distinct regulatory mechanisms in different phases of disease progression [105]. Thus, future research should prioritize the elucidation of these stage-dependent autophagic alterations and invest in the development of reliable in vivo tools to measure autophagy flux. Such insights are essential for designing therapeutic strategies that target autophagy appropriately at different stages of AD pathogenesis.

### 6.4. Axonal Transport and Autophagic Vacuole Accumulation

In healthy neurons, AVs are retrogradely transported along axons to the soma, where they undergo degradation [106]. However, in AD, this transport is disrupted, leading to the pathological accumulation of AVs in axons and dendrites [107]. Tau protein, which is primarily localized to axons, plays a pivotal role in stabilizing microtubules and facilitating AV trafficking [108]. However, in AD, tau becomes hyperphosphorylated, disrupting microtubule dynamics and contributing to both defective AV transport and broader autophagic dysfunction. Hyperphosphorylated tau can also impede autophagosome-lysosome fusion and promote the accumulation of toxic protein aggregates [109].

Tau is efficiently degraded through the autophagy pathway and is also known to modulate autophagy itself [97]. Disruption of the autophagy–lysosome system can lead to tau oligomerization and aggregation, processes which are alleviated by enhanced autophagy [110]. Furthermore, tau pathology may induce lysosomal abnormalities, suggesting a reciprocal relationship between tau hyperphosphorylation and autophagy failure [111]. Tau also plays a role in autophagosome maturation and retrograde trafficking by promoting microtubule stability and facilitating fusion with lysosomes [110]. While tau deficiency appears to confer protection against Aβ toxicity, the presence of tau is essential for mediating Aβ-induced neurotoxicity in Alzheimer’s disease models [112].

Conversely, other evidence suggests that lysosomal protease dysfunction, rather than tau abnormalities, may be the primary driver of axonal transport deficits [113]. Further studies are needed to delineate the precise molecular defects contributing to AV trafficking failure in AD. Notably, PS1 is essential for lysosomal acidification and autophagosome-lysosome fusion [114]. Fibroblasts from individuals with familial AD-associated PS1 mutations show AV accumulation and impaired turnover of aggregated proteins [115]. Some data suggest that the endolysosomal dysfunction in PS1-deficient cells arises from calcium homeostasis disruption, rather than proton pump defects [116].

### 6.5. Autophagy–Lysosome Pathway Disruption: Mechanistic Insights

Mechanistic studies have shown that autophagy–lysosome dysfunction in AD is linked to impaired proteolysis within autolysosomes [117]. Both human and mouse AD brains exhibit autolysosomes filled with incompletely degraded cargo, such as LC3-II, ubiquitinated proteins, and Aβ. Inhibition of lysosomal cathepsins, key proteases for cargo degradation, results in similar AVs accumulation, underscoring the importance of these enzymes [118].

Mutations in PS1 may further impair lysosomal calcium homeostasis, thereby compromising autophagosome-lysosome fusion [93]. Additionally, defects in lysosomal acidification have been associated with cholesterol accumulation in AD neurons, which inhibits hydrolase activity and impairs autophagosome clearance [119]. These converging defects result in a collapse of proteostasis, driving neurotoxicity and disease progression. Therapeutic strategies aimed at restoring lysosomal pH and modifying lipid composition may represent promising avenues for reversing autophagic failure in AD. Additionally, beyond classical molecular frameworks, alternative perspectives such as the biostructural theory proposed by Eugen Macovschi conceptualize living matter as a structurally organized, non-equilibrium system whose functional decline manifests through alterations in structural integrity and energy dissipation [120]. Although less explored in mainstream biomedical literature, this outlook provides a broader view of cellular degeneration. Furthermore, even subtle decreases in intracellular pH can significantly influence lysosomal activity, enhancing protease function and modulating the dynamics of autophagic degradation [121,122]. Such physicochemical parameters may alter the threshold for proteostasis and neurodegeneration, adding an important layer of complexity to our current mechanistic understanding.

Moreover, while hyperphosphorylated tau has been mechanistically linked to impaired autophagosome-lysosome fusion and aggregate accumulation, alternative frameworks suggest that tau hyperphosphorylation may also reflect deeper disruptions in cellular structure and energy regulation [123]. According to Eugen Macovschi’s biostructural theory, degeneration involves a breakdown of the living biostructure, which destabilizes intracellular organization and may lead to uncontrolled ATP-dependent reactions such as aberrant phosphorylation [120]. This perspective suggests that tau pathology might not only be a molecular driver but also a secondary manifestation of broader biostructural failure within neurons.

### 6.6. Aβ Secretion and Autophagy Crosstalk

APP-derived metabolites can accumulate intracellularly by inhibiting autophagy before lysosomal fusion. The genetic deletion of autophagy-related genes such as ATG5 or ATG7 recapitulates AD-like pathology, demonstrating the importance of intact autophagy in preventing Aβ accumulation [120]. Autophagy plays a dual role in AD: it degrades Aβ and may also contribute to its production and secretion under pathological conditions [81,124].

While autophagy typically promotes Aβ clearance, emerging evidence indicates that it can also facilitate extracellular Aβ release, contributing to plaque formation [125]. The genetic deletion of autophagy components exacerbates intracellular Aβ buildup, highlighting the critical balance between degradation and secretion. Aβ itself may regulate autophagy in a feedback loop, inducing autophagic responses while simultaneously disrupting autolysosomal membrane integrity, leading to degradation failure [125].

Autophagy activation by Aβ42 appears to be initially protective; however, prolonged activation can shift to a pro-death role, contributing to neuronal degeneration [126]. Furthermore, autophagy-derived exosomes may serve as vehicles for intercellular transmission of Aβ and tau, thereby facilitating the spatiotemporal spread of pathology across brain regions [106]. Targeting the crosstalk between autophagy, Aβ metabolism, and intercellular signaling may offer novel therapeutic strategies.

### 6.7. The Liver’s Role in Autophagy and AD Pathogenesis

Emerging evidence suggests that the liver contributes to cerebral Aβ burden, acting both as a site of peripheral Aβ clearance and a potential source of circulating Aβ [127]. Mutations in APP, PS1, and PS2, key regulators of Aβ production, are associated with increased Aβ42 formation and deposition in AD mouse models [127]. Surprisingly, PS2 activity is heritable and liver-specific in mice, suggesting that hepatic dysfunction may contribute to cerebral Aβ accumulation [128].

**Figure 4:**
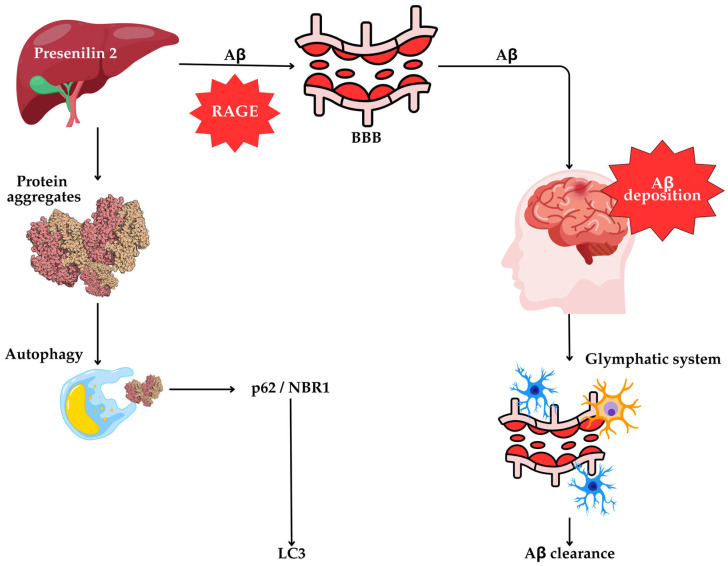
Presenilin 2 (PS2)-mediated amyloid beta (Aβ) synthesis in the liver and its contribution to cerebral amyloid burden. PS2 activity in the liver promotes the synthesis of Aβ, which can enter the brain via the blood–brain barrier (BBB) through the receptor for advanced glycation end-products (RAGE), contributing to cerebral Aβ accumulation. Clearance of Aβ from the brain occurs via the glymphatic system, the brain’s waste removal pathway. Intracellularly, misfolded protein aggregates, including Aβ, are targeted for degradation by autophagy. The cargo adaptor proteins p62 (also known as Sequestosome 1 or SQSTM1) and NBR1 bind ubiquitinated protein aggregates and facilitate their delivery to autophagosomal membrane proteins such as microtubule-associated protein 1A/1B light chain 3 (LC3), enabling lysosomal degradation and maintaining proteostasis.

Aβ can reach the brain through several pathways, including blood–brain barrier (BBB) transport and the glymphatic system, which is mediated by brain low-density lipoprotein receptor-related protein 1 (LRP1) [129]. Conversely, the receptor for advanced glycation end-products (RAGE) facilitates Aβ influx across the BBB, promoting cerebral accumulation [127] (Figure 4).

Additionally, autophagy plays a central role in hepatic protein quality control, clearing misfolded and aggregated proteins that accumulate during stress or metabolic dysfunction [129,130]. In Atg7 knockout mice, the loss of hepatic autophagy leads to the accumulation of ubiquitinated protein aggregates, peroxisomes, and defective mitochondria, ultimately causing hepatomegaly and liver damage [131]. The autophagy receptors p62/SQSTM1 and NBR1 mediate the degradation of ubiquitinated proteins, and their co-accumulation upon autophagy inhibition underscores their importance in selective autophagy [132,133,134].

In the context of alcoholic liver disease, p62 is found in Mallory bodies, suggesting a role in inclusion body formation [116]. Liver-specific deletion of Atg7 results in p62 accumulation, which alters gene expression, enhances nuclear factor kappa B (NF-κB) activity, increases oxidative stress, and promotes tumorigenesis [135]. Moreover, p62 can also facilitate caspase-8 activation via aggregation, further linking autophagy dysfunction to apoptotic signaling [136].

Thus, hepatic autophagy is a crucial physiological process for maintaining proteostasis and systemic homeostasis. Its impairment may influence both peripheral and central Aβ dynamics, and restoring hepatic autophagy could represent an innovative therapeutic approach in AD. However, further research is warranted to clarify the mechanistic links and evaluate interventional strategies.

Importantly, clinical evidence supports a link between hepatic dysfunction and cognitive decline. A UK-based cohort (over 400,000 participants) found that markers of liver dysfunction including elevated aspartate aminotransferase (AST), gamma-glutamyl transpeptidase (GGT), AST/ALT ratio, and hypoproteinemia were significantly associated with the development of dementia, which includes cognitive decline and volumetric loss in hippocampal and subcortical structures [137]. Similarly, a smaller retrospective cohort of 4582 individuals with metabolic-associated steatotic liver disease (MASLD) showed a nearly threefold increased risk of AD compared to controls (RR 2.80; 95% CI 1.79–4.38) [138]. Large-scale studies also found that severe liver fibrosis/cirrhosis was associated with a 9–18% increased risk of dementia [139]. Finally, up to 10% of patients initially diagnosed with dementia may have reversible cognitive decline due to hepatic encephalopathy from undiagnosed cirrhosis [140]. Together, these data support a potential causal and bidirectional relationship between liver health and AD risk, reinforcing the systemic nature of proteostatic and metabolic dysfunction in neurodegeneration.

### 6.8. Relative Contributions of Autophagic Pathways to AD Pathogenesis

Although previous sections describe macroautophagy, selective autophagy, and CMA based on their mechanisms, it is crucial to recognize that not all autophagic pathways contribute equally to AD pathogenesis. Emerging evidence suggests that specific autophagy types, particularly macroautophagy and CMA, play dominant roles in the development and progression of AD.

Macroautophagy, particularly mitophagy, is a crucial pathway for clearing aggregated proteins and dysfunctional organelles. In AD, impairment of macroautophagy has been well documented and is linked to the accumulation of autophagic vacuoles, misfolded tau and Aβ aggregates, and defective axonal transport [141,142]. Specifically, mitophagy dysfunction causes oxidative stress and energy deficits in neurons, which accelerates neurodegeneration [143,144]. Recent reviews also highlighted that research in transgenic AD models has demonstrated that enhancing macroautophagy can decrease Aβ accumulation and enhance synaptic function [145].

CMA also contributes to proteostasis by targeting specific cytosolic proteins, including tau, for lysosomal degradation. In AD, CMA dysfunction has been linked to reduced expression and impaired assembly of the lysosomal receptor LAMP-2A, resulting in inefficient clearance of tau and other neurotoxic proteins [146]. Notably, the ApoE4 isoform, a major genetic risk factor for sporadic AD, has been shown to inhibit CMA by disrupting lysosomal stability and receptor availability [147,148]. Thus, CMA dysfunction not only contributes to tauopathy but may also interact with genetic vulnerabilities in AD [146].

In contrast, the roles of microautophagy and endosomal microautophagy in AD remain less well-defined. While these pathways participate in vesicle turnover and cytoplasmic content recycling, their direct involvement in AD-related proteinopathies is still under investigation [149]. Nevertheless, alterations in endo-lysosomal trafficking observed in AD suggest that these processes may play indirect or modulatory roles in disease progression [150]. In summary, the macroautophagy–lysosomal system and CMA are the most critically involved autophagic pathways in AD pathophysiology. Understanding their differential roles is essential for developing targeted therapeutic strategies aimed at restoring autophagic flux and proteostasis in affected neurons.

## 7. Future Research

Despite significant advancements in understanding the pathological mechanisms of AD, the role of peripheral organs, particularly the liver, in modulating cerebral Aβ burden remains an underexplored but potentially transformative area. Future research must investigate the functional relationship between hepatic autophagy and brain Aβ homeostasis, aiming to delineate the molecular and systemic pathways that link liver metabolism to neurodegeneration. This could be achieved through the use of sophisticated in vivo models, such as liver-specific autophagy knockout or overexpression systems in genetically engineered AD mice. These models would allow for a temporal and spatial dissection of how altered hepatic autophagic flux influences Aβ production, clearance, and deposition in the brain [151,152,153].

Importantly, while hepatic autophagy is known to play a role in proteostasis and systemic metabolic regulation, its direct impact on circulating Aβ levels and subsequent brain pathology has not been thoroughly quantified. The hypothesis that impaired hepatic clearance contributes to cerebral Aβ accumulation represents a paradigm shift from brain-centric AD models to a more systemic disease framework. Such an approach is not only biologically plausible given the liver’s central role in metabolic detoxification and protein clearance, but also therapeutically attractive, as hepatic interventions may circumvent the BBB, which remains a formidable challenge in AD drug delivery [154].

Several treatments have been studied to activate autophagy, offering promising opportunities for AD treatment. For example, rapamycin, an mTOR inhibitor, enhances autophagy, promoting the clearance of Aβ and tau aggregates in AD models, which may offer promising outcomes [155]. Similarly, resveratrol activates SIRT1, upregulating autophagy, reducing Aβ accumulation, and neuroinflammation [156]. Moreover, curcumin induces autophagy via AMPK activation and mTOR inhibition and has shown potential in reducing Aβ plaque burden and warrants deeper investigation [157]. Valproic acid and suberoylanilide hydroxamic acid (SAHA), compounds that inhibit HDAC, can improve autophagy by modifying histone acetylation, thereby enhancing protein clearance and potentially treating neurodegenerative diseases [158].

Besides pharmaceutical treatments, natural stimuli provide a non-invasive method for activating autophagy, which may yield potential benefits in Alzheimer’s disease. Physical activity has been demonstrated to stimulate autophagy in peripheral tissues, including the liver, muscle, and brain. This contributes to enhanced Aβ clearance. Interestingly, exercise also enhances neural synaptic plasticity, fosters adult neurogenesis, mitigates cognitive loss associated with aging, and postpones the onset of neurodegenerative disorders [159]. Caloric restriction similarly enhances autophagy, a cytoplasmic recycling process, by decreasing protein acetylation [160]. Thus, these findings highlight the therapeutic significance of lifestyle modifications in regulating autophagy for neuroprotection.

To this end, future research should also focus on the development of pharmacological agents or gene therapy techniques aimed at selectively enhancing hepatic autophagy. Small molecule modulators of autophagy regulators (e.g., TFEB activators, mTOR inhibitors) or targeted delivery of autophagy-related genes (such as Atg7, p62/SQSTM1, and NBR1) via liver-specific vectors could be explored. Such approaches should be rigorously tested in preclinical models to evaluate their capacity not only to upregulate autophagy but also to reduce Aβ burden and improve cognitive outcomes.

Another important research avenue involves the identification of reliable systemic biomarkers of autophagy. Most current autophagy studies rely on brain tissue analysis, which is not feasible in living patients. Peripheral blood-based or liver-derived biomarkers (e.g., circulating p62, LC3-II levels, exosomal Aβ or tau) could enable early detection, risk stratification, and dynamic monitoring of disease progression or therapeutic response [161]. However, distinguishing disease-specific autophagy signals from those associated with normal aging or comorbidities remains a major challenge and necessitates longitudinal human cohort studies.

Critically, any proposed intervention that targets peripheral autophagy must also consider potential trade-offs and unintended consequences. For instance, excessive autophagic activation could lead to hepatocellular stress or dysfunction, particularly in aging or metabolically compromised individuals [162]. The efficiency of autophagy-stimulating therapies may differ according to the stage of AD and the age of the individual, as older adults often have diminished autophagy, which may impair treatment effects. Furthermore, systemic modulation of autophagy might influence immune responses, which are increasingly recognized as key players in AD pathophysiology.

Adding to these concerns, one of the notable consequences of increased autophagic activity is the initiation of autophagic cell death, referred to as autosis. This is a non-apoptotic autophagy-dependent cell death, which is a unique morphological feature that depends on the cellular Na^+^, K^+^-ATPase. Autosis can occur during starvation, autophagy-inducing peptide treatment, and cerebral hypoxia-ischemia, leading to cellular self-destruction. Its discovery could stimulate molecular mechanisms, understand autophagic cell death’s role, and provide novel therapeutic targets [163].

In summary, targeting autophagy beyond the central nervous system, particularly in the liver, represents a promising but complex frontier in AD research. Future studies should aim to integrate multi-organ models, systems biology approaches, and translational pipelines to fully harness the therapeutic potential of peripheral autophagy modulation. This shift toward a holistic, systems-level understanding of AD could pave the way for more effective and accessible interventions in the prevention and management of this devastating neurodegenerative disease.

## 8. Conclusions

Autophagy is a fundamental cellular process that maintains homeostasis by degrading misfolded proteins and damaged organelles. In AD, the disruption of autophagy contributes to hallmark pathological features, including Aβ accumulation, tau hyperphosphorylation, and progressive neurodegeneration. While much of the existing literature has focused on neuronal autophagy and its dysregulation within the brain, emerging evidence highlights a critical and previously underappreciated role for hepatic autophagy in modulating systemic Aβ dynamics. This review underscores the liver’s involvement in peripheral Aβ metabolism, possibly through presenilin-mediated pathways and autophagy-dependent clearance mechanisms. Aberrant liver function, particularly impaired autophagic flux, may lead to increased circulating Aβ levels and contribute to its accumulation in the brain. The recognition that the liver is not merely a bystander but an active player in AD pathophysiology challenges the traditional neurocentric view of the disease and opens up new avenues for therapeutic intervention. By integrating central and peripheral autophagy into the broader framework of AD pathology, we advance a more systemic perspective of the disease. This conceptual shift not only broadens our understanding of disease mechanisms but also suggests novel treatment strategies, especially those aimed at restoring hepatic autophagic function, to reduce brain Aβ burden. Continued research in this direction could lead to the development of multi-organ targeted therapies and innovative biomarker platforms, potentially transforming the diagnosis and treatment of AD.

## Data Availability

Not applicable.

## Abbreviations

The following abbreviations are used in this manuscript:

AAA-ATPase VCP/p9	ATP-driven chaperone valosin-containing protein
AD	Alzheimer’s disease
AMPK	AMP-activated protein kinase
ApoE4	Apolipoprotein E4
APP	Amyloid precursor protein
ATG	Autophagy-related genes
AVs	Autophagic vacuoles
Aβ	Amyloid-beta
BBB	Blood–brain barrier
BECN	Beclin
BNIP3L	BCL2/adenovirus E1B 19 kDa protein-interacting protein 3-like
CCPG1	Cell cycle progression gene 1
CMA	Chaperone-mediated autophagy
DFCPI	Double FYVE-containing protein 1
EF1α	Elongation factor-1α
ESCRT	Endosomal sorting complex required for transport
FAM134B	Family with Sequence Similarity 134 Member B
FIP200	FAK family kinase-interacting protein of 200 kDa
GFAP	Glial fibrillary acidic protein
HIF-1α	Hypoxia-inducible factor-1α
HSC70	Cytosolic chaperone heat shock cognate 71-kDa protein
JLK	UNC-51-like autophagy-activating kinase
LAMP-2A	Lysosome-associated membrane protein type 2A
LANDO	LC3-associated endocytosis
LAP	LC3-associated phagocytosis
LC3	Microtubule-associated protein 1A/1B light chain 3
LE/MVBs	Late endosomes/multivesicular bodies
LLOMe	L-leucyl-L-leucine methyl ester
LRP1	Low-density lipoprotein receptor-related protein 1
mTORC1	Mechanistic target of rapamycin complex 1
NDP52	Nuclear dot protein 52 kDa
NF-κB	Nuclear factor kappa B
NFAT	Nuclear factor of activated T-cells
NIX	NIP3-like protein X
NRF2	Nuclear factor erythroid 2-related factor 2
OPTN	Optineurin
PARP	Poly(ADP-ribose) polymerase
PAS	Phagophore assembly site
PE	Phosphatidyl-ethanolamine
PEX3	Peroxisomal biogenesis factor 3
PINK1	PTEN-induced kinase 1
PS-1 or 2	Presenilin-1 or 2
PtdIns3P	Phosphatidylinositol 3-phosphate
RAGE	Receptor for advanced glycation end-products
RARα	Retinoic acid receptor alpha
SNARE	Soluble N-ethylmaleimide-sensitive-factor attachment protein receptor
SQSTM1	Sequestosome 1
STX17	Syntaxin 17
TAX1BP1	Tax1 (Human T-cell Leukemia Virus Type I) binding protein 1
TBK1	Tank-binding kinase 1
TPD52	Tumor protein D52
TEX264	Testis expressed 264
TFEB	Transcription factor EB
TLR	Toll-like receptors
TNKS1	Tankyrase 1
UPS	Ubiquitin-proteasome system
VHL	von Hippel–Lindau protein
VPS34	Vacuolar protein sorting 34
WIPI	WD repeat domain, phosphoinositide interacting
